# Identification of Protein Partners in Mycobacteria Using a Single-Step Affinity Purification Method

**DOI:** 10.1371/journal.pone.0091380

**Published:** 2014-03-24

**Authors:** Przemysław Płociński, Daniel Laubitz, Dominik Cysewski, Krystian Stoduś, Katarzyna Kowalska, Andrzej Dziembowski

**Affiliations:** 1 Institute of Biochemistry and Biophysics, Polish Academy of Sciences, Warszawa, Poland; 2 Department of Genetics and Biotechnology, Faculty of Biology, University of Warsaw, Warsaw, Poland; University of Delhi, India

## Abstract

Tuberculosis is a leading cause of death in developing countries. Efforts are being made to both prevent its spread and improve curability rates. Understanding the biology of the bacteria causing the disease, *Mycobacterium tuberculosis* (*M. tuberculosis*), is thus vital. We have implemented improved screening methods for protein–protein interactions based on affinity purification followed by high-resolution mass spectrometry. This method can be efficiently applied to both medium- and high-throughput studies aiming to characterize protein–protein interaction networks of tubercle bacilli. Of the 4 tested epitopes FLAG, enhanced green fluorescent protein (eGFP), protein A and haemagglutinin, the eGFP tag was found to be most useful on account of its easily monitored expression and its ability to function as a simultaneous tool for subcellular localization studies. It presents a relatively low background with cost-effective purification. RNA polymerase subunit A (RpoA) was used as a model for investigation of a large protein complex. When used as bait, it co-purified with all remaining RNA polymerase core subunits as well as many accessory proteins. The amount of RpoA strongly correlated with the amount of quantification peptide used as part of the tagging system in this study (SH), making it applicable for semi-quantification studies. Interactions between the components of the RpoA-eGFP protein complex were further confirmed using protein cross-linking. Dynamic changes in the composition of protein complexes under induction of UV damage were observed when UvrA-eGFP expressing cells treated with UV light were used to co-purify UvrA interaction partners.

## Introduction

*Mycobacterium tuberculosis*, the causative agent of tuberculosis (TB), is a deadly human pathogen, and it has emerged as an epidemic in many developing countries. The latest WHO report states that 8.6 million new cases of TB occurred in 2012. Moreover, TB threatens the lives of HIV-positive individuals, killing 3,20,000 of HIV-positive patients in 2012. Emergence of multidrug-resistant (MDR)-TB and totally drug-resistant (TDR)-TB strains has created an urgent need for profound investigation of the tubercle bacilli's physiology and pathogenicity. Understanding its biology is fundamental for developing new effective strategies to combat TB. Genomic and proteomic methods are being utilized to broaden this knowledge and to understand the network of protein–protein interactions for a variety of organisms, including pathogenic bacteria, in order to elucidate the regulation and dynamics of important cellular functions and processes including DNA replication, transcription and virulence.

Recent proteogenomic analysis identified 3,176 proteins from *M. tuberculosis*, representing approximately 80% of its total predicted number of genes [1]. Protein–protein interaction studies, which are crucial for understanding many biological processes, are not being performed to a satisfactory extent at present. Most often, protein–protein interactions are determined by researches only for very specific biological processes, and global protein–protein interaction networks of only few model organisms have been investigated on the basis of medium- or high-throughput experiments. These organisms include *Mycoplasma pneumoniae* [2], *Helicobacter pylori* [3], *Saccharomyces cerevisiae* [4], [5] and *Drosophila melanogaster* [6]. Analysis of protein–protein interactions in human pathogens will ultimately aid in better understanding of their biology and aid therapeutic discovery.

The most comprehensive protein–protein interaction network for the *M. tuberculosis* proteome was built using the bacterial two-hybrid (B2H) system [7]. The B2H and yeast two-hybrid (Y2H) systems are the most commonly used tools to study protein–protein interactions. They are powerful techniques, but intrinsically carry major limitations. A large caveat is that the screening is far from physiological conditions, with a high rate of false-positive and -negative results [8]. To increase the number of genes encoding potentially interactive protein partners, the two-hybrid system was modified to incorporate 3 different genes, allowing independent expression and interaction of mycobacterial proteins in *Escherichia coli* (*E. coli*). This three-hybrid system was used for the RD1 complex of *M. tuberculosis* [9]. However, this method can decipher only tri-protein complexes, establishing that its reliability does not reach global and complex protein–protein interactions and it must be supported by other techniques. There is also a dedicated two-hybrid assay, called the mycobacterial protein fragment complementation (M-PFC) assay, which is based on reconstitution of murine dihydrofolate reductase and allows investigation of protein–protein interactions in *M. smegmatis* host. This method presents a clear advantage of studying protein complex formation under physiological conditions and was successfully implemented both for soluble as well as for membrane proteins [10], [11]. In a different study, computer analysis of the interactome (derived from the STRING 8.0 database) was used to analyse communication between a drug environment and proteins involved in resistance to them to identify the most plausible paths that triggered the emergence of drug resistance [12].

Here we propose a single-epitope affinity purification (AP) technique combined with LC–MS/MS as a screening method for studying protein–protein interactions specifically in *Mycobacterium*. To determine the most efficient epitope, we designed 4 constructs containing 4 different fusion tags to be tested with targeted proteins. For further experiments, we selected FLAG, haemagglutinin (HA), protein A (ProtA) and enhanced green fluorescent protein (eGFP) epitopes. We employed a localization and affinity purification (LAP) method coupled with tandem mass spectrometry (LC–MS/MS), an efficient tool to investigate protein–protein interactions in living cells under close-to-physiological conditions [13]. This method typically produces a number of qualitative and descriptive results. Moreover, we provide evidence that chemical cross-linking followed by MS is applicable to native mycobacterial complexes to decipher direct contact sites between identified subunits.

The most sensitive and reliable tag for protein–protein interaction and protein complex analysis in mycobacteria was employed to determine subunits of the evolutionary conserved and stable DNA-dependent RNA polymerase that is well described in other microorganisms. We also used this tag to describe, for the very first time in *Mycobacterium*, dynamic changes in UvrABC DNA repair protein complex composition after UV irradiation. We strongly believe that the experimental system along with computational and informatics strategies [reviewed recently by Nesvizhskii [14]] holds the potential to aid in understanding the biology of *M. tuberculosis*. It will also assist in deciphering cross-talk between the pathogen and its host and may potentially elucidate weak points of the interaction against which drugs may be targeted.

## Materials and Methods

### Vectors and constructs

**Figure 1:**
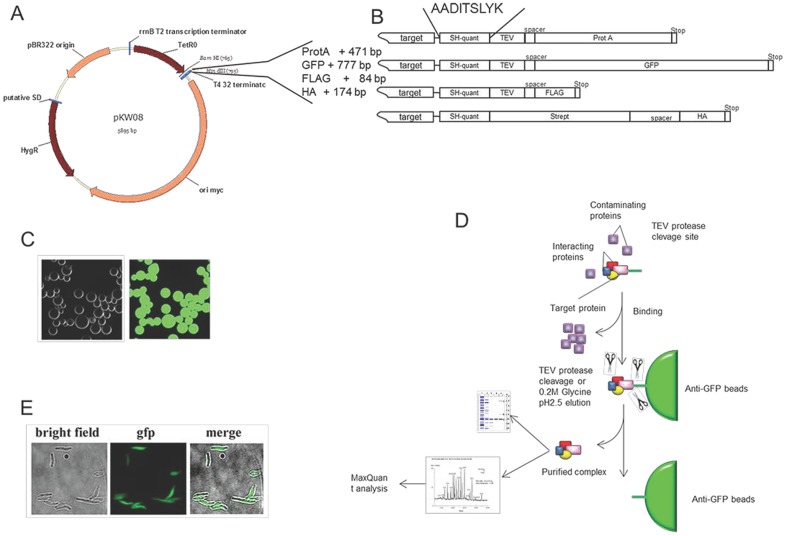
Schematic of the strategy used to purify protein complexes and identify protein–protein interactions in mycobacteria. (A) For expression of selected bait, pKW08-derived plasmids were engineered. Genes selected for further tests were cloned into the constructed vectors, allowing fusion with specific tags. (B) To minimize differences between tags, all tags were designed in a similar manner, containing the SH-quant, a cleavage site for TEV protease, a spacer and a single specific epitope terminating with a stop codon. Recombinant proteins were expressed in mycobacterial cells and the bait was purified on an epitope-specific resin. (C) Anti-GFP nanobodies prepared for this work were immobilized on activated Sepharose beads, and eGFP binding was examined by microscopy. (D) The overview of the purification procedure followed by LC–MS/MS and protein identification by MaxQuant software. (E) eGFP can be used to visualize the subcellular localization of a target protein.

We designed a suite of vectors with identical backbones on the basis of a pKW08 vector [15]. Four different epitopes containing HA, FLAG, ProtA or eGFP were selected. The gene encoding the protein of interest was separated from the epitope sequence by the cassette encoding a tobacco etch virus (TEV) protease cleavage site, followed by an SH-quant peptide and a 6-nucleotide spacer (Fig. 1A and 1B). This design allows our cassettes to be used for MS-based qualitative analysis and absolute quantification of protein complex components by adding defined amounts of an isotope-labelled heavy version of the SH-quant peptide {AADITSLY[Lys(13C6; 15N2)]; SH-quant*} to the sample [16]. The amino acid sequence of each tag was back-translated into the DNA sequence using a *M. smegmatis* codon usage table, and the nucleotide sequences of the designed tags were submitted for commercial synthesis (GenScript, USA; Integrated DNA Technology, USA). Respective sequences were introduced into the modified pKW08 plasmid to produce vectors suitable for tagging genes of interest, as described in the cloning section.

### Cloning

Our cloning strategy is based on a sequence- and ligation-independent cloning (SLIC) method [17]. In brief, all inserts intended for cloning were amplified using a pair of 50 nucleotide primers, with the first 30 nucleotides overlapping with the vector's compatible ends and the other 20 nucleotides complementing the insert. First, the pKW08 vector was linearized with BamHI and HindIII restriction enzymes according to the manufacturer's protocols (DoubleDigest tool, Fermentas, Thermo Scientific). The primary insert containing the SH-quant and epitope tags was cloned into the vector using the universal forward primer F1 and a tag-specific primer. The BamHI and HindIII sites were restored at the 5′-end of the tag. Subsequently, the pKW08 vector containing the respective tag was prepared by BamHI/HindIII restriction digestion. Genes encoding bait proteins for protein complex purification were amplified via PCR using the appropriate primer pairs. The 18-nucleotide sequence containing the Shine–Dalgarno box (GGAGGAG) was introduced into the forward primer sequence upstream of the start codon of the bait protein sequence. Full primer sequences are presented in Table S1. As part of the 7FP collaborative project *SysteMTb* founded by EC, we can access the Gateway Entry Clone Library (Pathogen Functional Genomics Resource Center, J. Craig Venter Institute, sponsored by NIAID), which comprises 3295 cloned ORFs from *M. tuberculosis* H37Rv supplemented with 430 unique cloned ORFs from *M. tuberculosis* CDC1551, for a total of 3725 validated entry clones. All entry clones are flanked with *att* sites, allowing design of universal primers for the entire library, where 30 nucleotides overlap with the vector's cloning compatible ends and 26 nucleotides can homologously recombine with the vector’s *att* sequences. When C-terminal tagging is required, the Shine–Dalgarno box can be constructed via 4 transition mutations (A→G) in the *attB1* site (gtacAAaAAagttgcccat → gtacGGaGGagttgcccat). N-terminal tagging requires only introduction of a stop codon between the 30 nucleotides and the *attB2* site.

The touchdown PCR (TD-PCR) protocol was used to increase specificity, sensitivity and yield of PCR products. Phusion High-Fidelity DNA Polymerase (Finnzymes, Thermo Scientific) was used to optimize insert amplification according to the manufacturer’s protocol (200 μM of each dNTP, 1× Phusion HF buffer, 0.5 μM of each primer, 0.02 U/μl Phusion DNA polymerase and 3%–8% DMSO). The annealing temperature was progressively lowered from 60°C to 50°C, decreasing 1°C every cycle. This temperature of 50°C was kept constant for the subsequent 25 cycles (98°C for 10 s, 50°C for 30 s and 72°C for 2 min). To ensure complete extension of the PCR products, the reactions were incubated for an additional 7 min at 72°C and then held at 4°C.

To clone an amplified insert, 100 ng of linearized vector and 200 ng of PCR product were mixed and treated with 0.5 U of T4 DNA polymerase (BioLabs) in buffer G (Fermentas) at room temperature (RT) for 10 min. The reaction was terminated by adding 1/10 volume of 10 mM dATP, followed by incubation on ice for 5 min. The annealing reaction was performed at 37°C for 30 min, followed by incubation on ice for transformation or storage at −20°C.

In general, 150 μl of chemically competent MH1 *E. coli* cells was transformed with the SLIC mixture. The bacterial cells were incubated with the SLIC mixture on ice for 30 min and then subjected to a heat shock at 42°C for 90 s in a water bath, followed by 2 min on ice. Next, the cells were incubated at 37°C in 850 μl of SOB medium for 1 h, permitting expression of transferred antibiotic resistance. The cells were pelleted, the medium was reduced to 100–200 μl and the cells were plated on LB plates containing hygromycin B (HygroGold, Invivogen) at a final concentration of 200 μg/ml.

### Bacterial strains and growth conditions

The mycobacterial strains used in this study included *M. smegmatis* mc^2^155 and *M. bovis* BCG Danish strain 1331 (SSI, Copenhagen, Denmark). The strains were cultured in Middlebrook 7H9 broth supplemented with sodium chloride, albumin, dextrose and catalase (ADC). For transformation of mycobacterial cells, appropriate parental strains were grown to exponential phase (OD_600_  =  0.6–0.8). The cells were then collected by centrifugation (4800 × *g*, 10 min, 4°C), washed 3 times with cold 10% glycerol and transformed via electroporation (25 μF, 1000 Ω, 2500 V). The cells were recovered in 5 ml of fresh media for 3 h at 37°C before plating. Transformants were selected on 7H11 solid media supplemented with ADC and hygromycin (50 μg/ml). To induce recombinant protein production, tetracycline was supplied in the growth media at a final concentration of 50 ng/ml. The cultures were grown in the presence of the inducer for 3 and 48 h for *M. smegmatis* and *M. bovis* BCG, respectively. Growth was monitored by optical density measurements at 600 nm.

### Protein complex purification

Mycobacterial cells were collected by centrifugation (15 min, 4800 × *g*, 4°C) and resuspended in 9 ml of cold sonication buffer containing 50 mM Tris (pH 8.0), 100 mM NaCl, 1 mM dithiotreitol (DTT, Sigma-Aldrich), 2 mM phenylmethylsulfonyl fluoride (PMSF, Sigma-Aldrich), 25 U/ml benzonase (Sigma-Aldrich,) and 0.5% Triton X-100 (Sigma-Aldrich). The buffer was supplemented with protease inhibitors (2 μM pepstatin A, 2 μg/ml chymostatin, 0.6 μM leupeptin, 1 mM benzamidine HCl and 0.1 M PMSF). The cells were transferred into 50-ml conical tubes and sonicated in the Diagenode sonication system in a cooled water bath (4°C) at high power (300 W) for 90 cycles of 45 s on and 30 s off. Cell debris was removed by centrifugation (20 min, 4800 × *g*, 4°C) and cleared whole cell lysates were transferred to new 15-ml conical tubes in which 40 μl of the tag-specific resin was added: anti-GFP Sepharose (prepared as described below) [18], anti-FLAG agarose (Sigma-Aldrich), anti-HA agarose (Sigma-Aldrich) or IgG Sepharose (GE Healthcare), for respective tagging systems. The samples were incubated for 2 h in a cold room with slow (6–8 rpm) end-to-end rotation. The beads were recovered on a polypropylene Poly-Prep chromatography column (Bio-Rad). The flow-through was collected, and for GFP-tagged samples, the fluorescence of GFP unbound to the beads was measured as described below. The columns with resin and captured proteins were washed 2 times with 10 ml of IPP150 buffer [10 mM Tris (pH 8.0), 150 mM NaCl and 0,1% Triton X-100 (Sigma-Aldrich)], followed by 2 washes with TEV buffer [10 mM Tris (pH 8.0), 150 mM NaCl, 0.5 mM EDTA and 1 mM DTT]. For tags containing sites recognized by TEV proteases (FLAG, eGFP and ProtA), 20 μl of TEV protease [cloned, expressed, purified and successfully used in our lab [19]] was added to 430 μl of TEV buffer and applied to the column to cleave off the bait protein from the beads, leaving the tags on the column. The TEV cleavage was performed at 4°C overnight. The purified proteins were collected into 1.5-ml Eppendorf tubes, and the columns were washed with TEV buffer to a final volume of 900 μl. HA-tagged proteins were eluted from the column by 400 μl of 0.2 M glycine–HCl (pH 2.5) into Eppendorf vials containing 50 μl of 1 M Tris buffer (pH 8.0) for neutralization. The final volume (900 μl) was adjusted with TEV buffer. The collected samples were mixed vigorously and divided into 2 equal parts. The bait protein with its interacting partners was precipitated by adding pyrogallol red–molybdate (PRM; 0.05 mM pyrogallol red, 0.16 mM sodium molybdate, 1 mM sodium oxalate, 50 mM succinic acid, pH 2.5; all from Sigma-Aldrich) reagent in 1/4 of the original volume and vigorously mixed for 30 s, followed by incubation at RT for at least 1 h. The precipitated proteins were centrifuged (25 min at 21000 × *g*, RT) and the supernatant was removed. One sample set was submitted for LC–MS/MS analysis and the second was resolved using SDS–PAGE. The overall workflow is presented in Figure 1D.

### UV damage induction

*M. bovis BCG* strain expressing the Rv1638/eGFP fusion protein was grown exponentially and induced with 50 ng/ml tetracycline, as described above. After induction, the cells were centrifuged (4800 × *g*, 10 min, RT), washed once with freshly prepared M9 minimal media and then centrifuged again. For each condition, the cell pellet from 500-ml cultures was suspended in 10 ml of minimal media, transferred to a Petri dish (ø 15 cm), placed on ice and irradiated with a Philips 15-W TUV lamp emitting UV at 254 nm with a final UV dose of 4.5 mJ/cm^2^ [20]. After exposure, the cells were immediately transferred to 37°C with moderate shaking and snap frozen in liquid nitrogen to halt UV damage recovery at times of 0, 1, 5, 15 and 30 min after exposure. Protein complexes were purified from each sample using the GFP trap and protocol described above.

### Anti-GFP Sepharose bead preparation

Anti-GFP nanobody-coupled Sepharose beads were specifically prepared for this work. To obtain antibodies against GFP, the amino acid sequence of Chain C of the GFP minimizer nanobody (NCBI Protein Database Accession Number: 3K1K_C) [21] was back-translated to its DNA coding sequence. Codons were optimized to ensure efficient expression in *E. coli*. A pelB leader sequence was introduced in front of the GFP minimizer for export to the bacterial periplasm and to ensure proper folding of the nanobody. The resulting DNA coding sequence was subsequently ligated in frame with the pET28PP vector, which allows the addition of a HisTag (6×) at the C-terminus for easier purification. The construct was transformed into *E. coli* BL21-CodonPlus-RIL and propagated overnight in LB liquid media containing kanamycin (50 μg/ml) and chloramphenicol (37.5 μg/ml) at 37°C. The bacterial cultures were diluted 1∶50 in autoinduction media (Formidium Super Broth Base including trace elements) used for large-scale protein expression and incubated at 18°C for 48 h with aeration in an orbital agitator (150 rpm). The cells were collected by centrifugation (10 min, 5000 × *g*, 4°C) and lysed by sonication (Branson 250, 40%, 15 min) in 20 mM Tris (pH 8.0)-based buffer containing 500 mM NaCl, 20 mM imidazole and 10 mM 2-mercaptoethanol. The crude cell lysate was clarified by centrifugation (45 min, 119046 × *g*, 4°C) and the supernatant was loaded onto a 5-ml Ni–NtA cartridge column (Qiagen). Unbound material was washed from the columns with 10 column volumes (CV) of lysis buffer followed by 10 CV of the same buffer with 1 M NaCl. Pure protein was eluted from the affinity column by using 5 CV of elution buffer of 500 mM NaCl and 600 mM imidazole. Affinity purification was followed by gel filtration with PBS buffer (containing 500 mM NaCl) using a Superdex 75 column (GE Healthcare). Subsequently, the purified GFP nanobodies were coupled with cyanogen bromide-activated Sepharose 4 Fast Flow (Sigma-Aldrich) beads. For coupling, Sepharose was washed with cold 1 mM HCl for 30 min (200 ml per 1 g of beads), followed by distilled water (10 bead volumes), and suspended in coupling buffer (PBS with 500 mM NaCl). The purified nanobodies were added to the solution for overnight coupling and stored in a cold room. The unbound ligand was washed away by several washes with coupling buffer, and unreacted groups on Sepharose were blocked by overnight incubation at 4°C with 200 mM glycine. The blocking agent was removed and the beads were extensively washed with coupling buffer. Finally, the beads were washed with 0.1 M NaAc (pH 4.0), followed by 500 mM NaCl and 100 mM Tris (pH 8.0), and stored in buffer containing 20 mM Tris (pH 8.0), 500 mM NaCl and 0.025% sodium azide as a preservative.

### Gel electrophoresis

The pelleted proteins were resuspended in loading buffer [10 μl of water, 4 μl of NuPage LDS sample buffer (Invitrogen) and 1 μl of 1 M DTT (Sigma-Aldrich)], boiled for 5 min and resolved on a 4%–12% gradient NuPage Bis–Tris gel (Invitrogen) using MES running buffer (Invitrogen) at 125 V. The PageRuler prestained protein ladder (Fermentas) was used as a molecular weight standard. The gels were stained with Coomassie for 2 h and destained overnight.

### Sample preparation, MS and peptide/protein identification

The protein pellets were dissolved in 50 μl of 100 mM NH_4_HCO_3_ and subjected to a standard procedure of trypsin digestion: the proteins were reduced with 10 mM DTT for 30 min at 56°C, alkylated with 55 mM iodoacetamide in darkness for 45 min at RT and digested overnight with 10 ng/μl trypsin. The resulting peptide mixtures were applied to RP-18 pre-columns of an HPLC system (Waters) using water containing 0.1% trifluoroacetic acid as the mobile phase, and transferred to a nano-HPLC RP-18 column (internal diameter: 75 μM, Waters) using an acetonitrile gradient (0%–35% ACN in 160 min) in the presence of 0.1% trifluoroacetic acid at a flow rate of 250 nl/min. The column outlet was directly coupled to the ion source of an Orbitrap Velos mass spectrometer (Thermo Scientific). A blank run ensured absence of cross-contamination from preceding samples.

The mass spectrometer was operated in a data-dependent mode to automatically switch between Orbitrap MS and LTQ–MS/MS acquisition. Survey full-scan MS spectra (from m/z 300 to 2000) were acquired in the Orbitrap with a resolution of R  =  15,000 at m/z 400 (after accumulation to a target of 1,000,000 charges in the LTQ). The method used allowed sequential isolation of the most intense ions (up to 5, depending on the signal intensity) for fragmentation on the linear ion trap using collision-induced dissociation at a target value of 30,000 charges. The target ions selected for MS/MS were dynamically excluded for 60 s. Chromatographic peak apex detection triggered data dependent scans (expected peak width: 5 s, minimal signal threshold: 10,000 counts) with phase method activated and triggering window set to 30%. General MS conditions were as follows: electrospray voltage, 1.8 kV; no sheath and auxiliary gas flow. The ion selection threshold was 10,000 counts for MS/MS, and an activation Q-value of 0.22 and activation time of 30 ms were also applied.

The raw files were processed, including peak list generation, using the MaxQuant (v1.3.0.5) computational proteomics platform and default parameters were used. The fragmentation spectra were searched using Andromeda search engine integrated into the MaxQuant platform against an *M. smegmatis* mc^2^155 protein database available at the CMR website (http://cmr.jcvi.org/tigr-scripts/CMR/CmrHomePage.cgi, 6878 entries, v15.1, Oct 15, 2004) or against an *M. bovis* BCG database (www.patricbrc.org, NC_008769, 3952 entries). The databases were modified in-house to contain randomized sequences of all entries to control for false-positive identifications during analysis using the Andromeda search engine. The error ranges for the first and main searches were 20 ppm and 6 ppm, respectively, with 2 missed cleavages. Carbamidomethylation of cysteines was set as a fixed modification, and oxidation and protein N-terminal acetylation were selected as variable modifications for database searching. The minimum peptide length was set at 7 aa. Both peptide and protein identifications were filtered at a 1% false discovery rate and were thus not dependent on the peptide score. Enzyme specificity was set to trypsin, allowing cleavage of N-terminal proline. A ‘common contaminants’ database (incorporated in MaxQuant software) containing commonly occurring contaminations (keratins, trypsin etc.) was employed during MS runs.

Bioinformatics analysis was performed using the Perseus tool (v1.3.0.4, Cox J., Max Planck, 2012). Contaminants and random protein identification were excluded. Proteins identified by less than 2 peptides were excluded from the results, except SH, the quantification peptide. Peptide and protein identification details, including scores, are provided in Tables S2 and S3.

### Protein cross-linking, mass spectrometric analysis and cross-link validation

For protein complex cross-linking, we selected the DNA-directed RNA polymerase, where the alpha subunit (rpoA, MSMEG_1524) was fused with the C-terminal GFP tag. The purification procedure was as described above, with the TEV cleavage buffer changed to a 10 mM HEPES (pH 8.0)-based buffer. The purified protein complexes eluted from the column were subjected to cross-linking. We used bis(sulfosuccinimidyl) suberate (BS3) as the cross-linker (Thermo Scientific), with an 8-carbon spacer arm (11.4 Å), according to the manufacturer’s protocol. Heavy (d4) and light (d0) versions of BS3 reagent were dissolved in DMSO and mixed at a 1∶1 ratio immediately before use. The d0/d4 mixture was used to induce stable and selective chemical cross-links between lysine (K) residues available on surfaces of purified proteins to fix potential interactions between protein partners. Next, 50 mM of the BS3 (d0/d4) mixture was added at a final concentration of 2 mM to purified proteins and incubated for 15 min at 4°C. The reaction was terminated by adding 10 μl of 3 M Tris solution (pH 8.0). The samples were precipitated with PRM, as described above. Subsequently, the proteins were digested overnight with 10 ng/ml trypsin (Promega) in 100 mM ammonium bicarbonate buffer at 37°C. The peptides were reduced in 10 mM DTT for 30 min at RT and alkylated in 55 mM iodoacetamide for 20 min at RT. Finally, trifluoroacetic acid was added at a final concentration of 0.1%.

To determine protein compositions of the cross-linked samples, we used MaxQuant software (as described above). To search for the cross-linked peptides, we used pLink (pFind Studio) [22]. The following parameters were used: precursor mass tolerance, 50 ppm; fragment mass tolerance, 20 ppm; cross-linker, light [d0]-BS3 and heavy [d4]-BS3 (cross-linking sites, K and protein N-terminus; xlink mass-shift, 138.0680796 and monolink mass-shift, 156.0786442); isotope shift, 4.0247 Da; fixed modification, C 57.02146 and enzyme, trypsin.

We used. mgf files (Mascot Generic Files generated from. raw files by Mascot Distiller) and a protein database containing proteins found in a preceding MaxQuant search. All looplinks and monolinks were excluded from our obtained results. Only inter- or intra-molecular cross-links were used for further analysis. Molecular graphics and analyses were performed with the UCSF Chimera package from the Resource for Biocomputing, Visualization and Informatics at the University of California, San Francisco (supported by NIGMS 9P41GM103311).

### Microscopic evaluation

The eGFP tag allows subcellular visualization of proteins of interest. *M. smegmatis* mc^2^155 expressing the RpoA-eGFP fusion protein was used as a model for testing GFP localization. The strain was grown in culture media described above to an OD_600_ of 0.6–0.8. Protein production was induced by adding 50 ng/ml tetracycline for 3 h. The cells were collected by centrifugation (15 min, 4800 × *g*, 4°C) and washed with PBS. As a counterstain, the nuclei were stained with 0.5 μg/ml DAPI for 10 min at RT. The cells were washed again to remove excess dye. The slides were mounted with fluorescent mounting medium (Dako). A IX81 fluorescence microscope (Olympus) fitted with a PLANAPO 100x/1.35 oil immersion objective and appropriate filter sets (Semrock) was used for bright-field and fluorescence microscopy, and images were acquired using an Orca R^2^ camera (Hamamatsu) and the Excellence software package. The images were processed using ImageJ 1.46r and Adobe Photoshop CS4 software.

### Fluorescence intensity measurement

To estimate the approximate efficiency of binding of eGFP-tagged bait proteins to the anti-GFP beads, the fluorescence intensity was measured. In brief, cell lysates derived from recombinant *M. smegmatis* strains expressing eGFP fusion proteins were prepared and pre-cleared by centrifugation and the flow-through after binding to the column was diluted 1∶1 in IPP150 buffer and transferred onto a 96-well black solid plate (Nunc, Thermo Scientific). Cell lysate from the *M. smegmatis* mc^2^155 parent was used as a background control. Lysates were prepared from approximately the same number of cells as measured by cell pellet weight. Fluorescence counts were measured using Beckman Coulter DTX 800/880 Multimode Detector and Multimode Detection software. Excitation at 485 nm and emission at 535 nm was used, with a data integration time of 1 s. The relative binding efficiency was calculated by dividing the fluorescence intensity of flow-through by the intensity of the lysate before binding to the column, multiplied by 100%.

## Results

Affinity tags serve as selective and efficient tools for protein purification and can be used for purification of native protein complexes. Of the many available tags, we examined 4 that we selected on the basis of predicted usefulness for high-throughput purification and analysis of protein complexes in mycobacteria. To simplify the method and ensure co-purification of the majority of philological interacting partners, including weak and transient interactions, we performed single-step purification. We selected FLAG [23], HA and the protein A IgG-binding domain [24], [25], which are all popular tags. Our fourth tag was based on eGFP protein and designed with the GFP-binding beads for these experiments. The tags we investigated are known to interact with appropriate/respective affinity resins coupled to specific antibodies. We restricted our study to affinity tags that could be eluted under relatively mild conditions, ensuring that we pull down protein complexes to analyse intra-cellular interactions. The FLAG, eGFP and ProtA tags contain the TEV protease cleavage site (Fig. 1), making protease cleavage a favourable method for elution of protein complexes. Because the HA tag was not provided with protease cleavage site, we could test an alternative elution method. We used the most effective mildly denaturing elution buffer 0.2 M glycine (pH 2.5). Applied low pH disrupts most antibody–antigen interactions and this elution method was particularly effective.

### Comparison of tags for protein complex purification

To compare efficiency and specificity of protein complex purification using the chosen tags, we selected 8 proteins from *M. smegmatis*. These proteins are implicated in different metabolic pathways (purine/pyrimidine metabolism, glycolysis/gluconeogenesis, pentose phosphate pathway) and fulfil varying cellular functions (e.g. RNA synthesis, glycolysis/gluconeogenesis and recombination). All selected genes encoding selected proteins are summarized in Table 1. Each gene was expressed in *M. smegmatis* in fusion with all 4 tags, giving a total of 32 combinations.

**Table 1:** List of selected targets.

Sample no.	Locus name	Gene definition	Gene length (nt)	Protein length (aa)
1	MSMEG0358	ribonucleoside-diphosphate reductase, beta subunit	963	320
2	MSMEG0752	fructose-bisphosphate aldolase, class II (*fbaA*)	1038	345
3	MSMEG1524	DNA-directed RNA polymerase, alpha subunit (*rpoA*)	1053	350
4	MSMEG1666	RNA polymerase sigma-70 factor	915	304
5	MSMEG2136	phosphoglucomutase, alpha-D-glucose phosphate-specific (*pgm*)	1635	544
6	MSMEG3021	AAA ATPase	1344	447
7	MSMEG3085	phosphoglycerate kinase (*pgk*)	1227	408
8	MSMEG3086	triosephosphate isomerase (*tpiA*)	786	261

**Figure 2:**
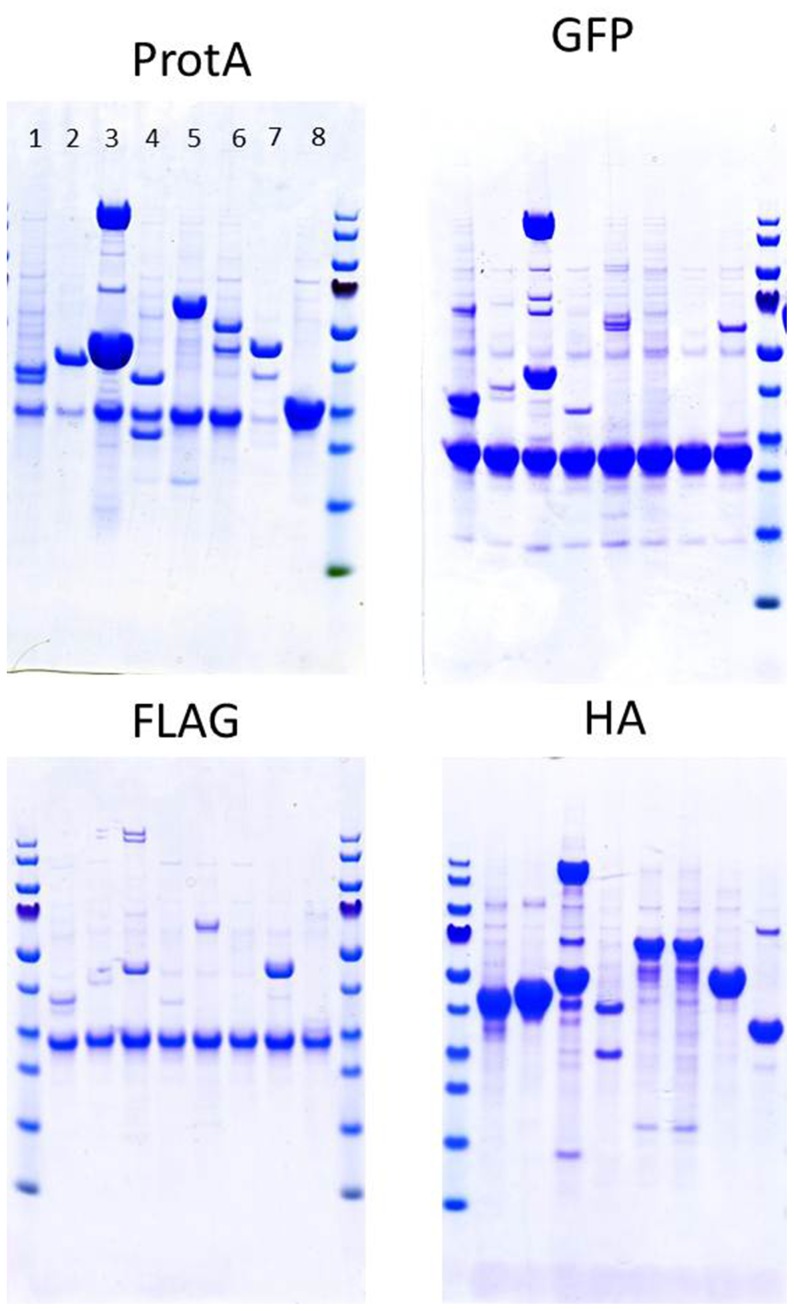
Polyacrylamide gel electrophoresis (Novex NuPage) of protein complexes purified using different tags on specific beads. For each tagging epitope, lanes 1– 8 represent protein complexes purified from *M. smegmatis* mc^2^155 expressing tagged proteins of interest. Details are listed in Table 1.

Each of the tagged genes was expressed and the resulting protein complexes were purified on a specific resin, followed by LC–MS/MS and computational analysis. Just prior to precipitation with PRM reagent, the samples were divided. Half of each sample was loaded on a Tris–glycine SDS–PAGE gel (Fig. 2) and the other half was subjected to LC–MS/MS analysis. All identifications, including the intensities calculated for each prey, are presented in Table S4.

**Figure 3:**
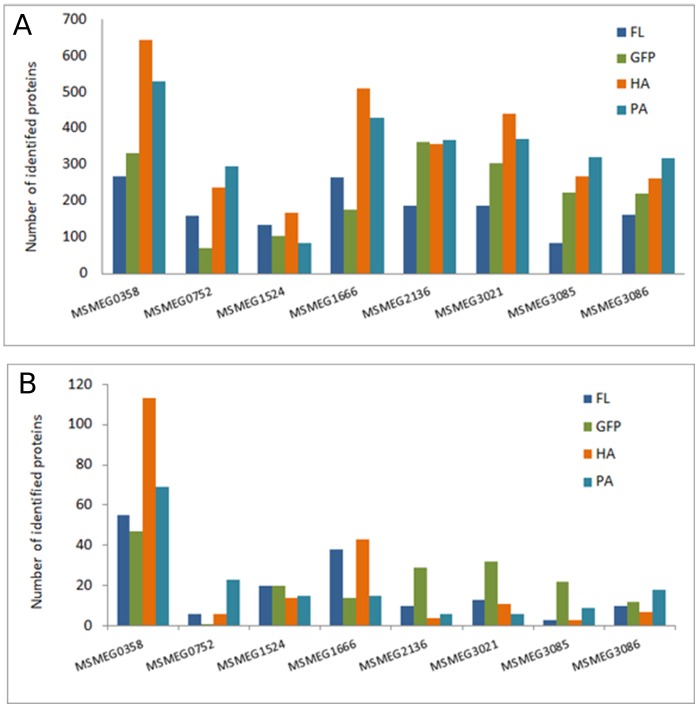
Number of proteins identified from a specific tag. (A) Total number of proteins identified by MaxQuant software and pulled down on FL, GFP, HA and ProtA resins. (B) Number of proteins purified exclusively with the target on specific beads. This set contains both prey specific for tagged protein as well as proteins not present in other purifications for the same resin.

LC–MS/MS experiments include high levels of contamination created mainly by non-specific interactions of proteins with the resin used for affinity purification. For complex peptide mixtures in cell lysates, co-elution may complicate biological evaluation of results. Of the most common contaminating proteins, we found chaperons, heat-shock proteins, ribosomal proteins and other proteins non-specifically bound to the purified protein complexes and the resin. To find a tag most useful in mycobacterial pull-downs and applicable for high-throughput experiments, we attempted to achieve a balance between the low number of total identified proteins (low background) without losing the real binding partners. Figure 3A shows the total number of identified proteins specific for the bait proteins and compares the number of obtained identifications with different tags purified on tag-specific resins. The lowest number of binding candidates was observed with FLAG and eGFP tags. HA and ProtA tags provided much longer lists of detected proteins (Table S1). Thus, based on the total number of identified proteins, FLAG and eGFP tags appear to be applicable with the lowest resin-specific background. We also discerned the number of exclusive proteins identified by MaxQuant software. The ‘exclusive proteins’ term represents all proteins specific for both the tag/resin and the bait, indicating a combination of real interactors and a protein background specific for the particular tag/resin. We observed that the number of purified proteins depends not only on the tag but also on the bait. For example, MSMEG0358, the beta subunit of ribonucleoside diphosphate reductase, was purified with the highest number of both total and exclusive identifications independent of the tag used. Of note, all tags selected for this work were cloned into and expressed from the same vector using identical induction conditions of the tetR08 promoter.

### Background evaluation

**Figure 4:**
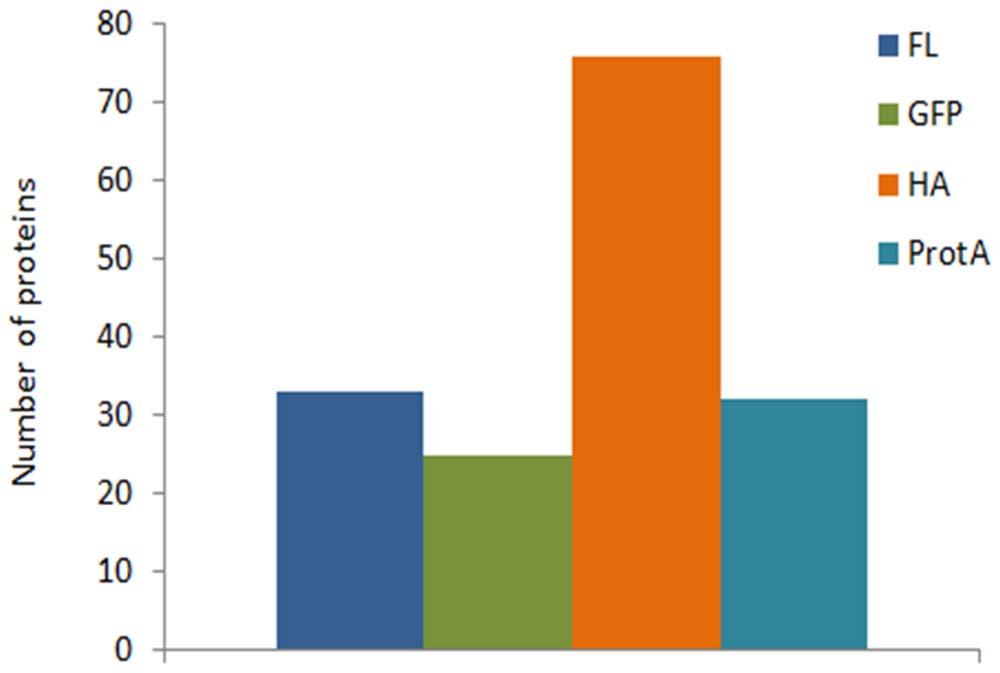
Number of identified proteins found as bead-specific background. These prey proteins were found in all 8 pull-downs, regardless of the target protein used as bait. The resin-specific background details are listed in Table S3.

**Figure 5:**
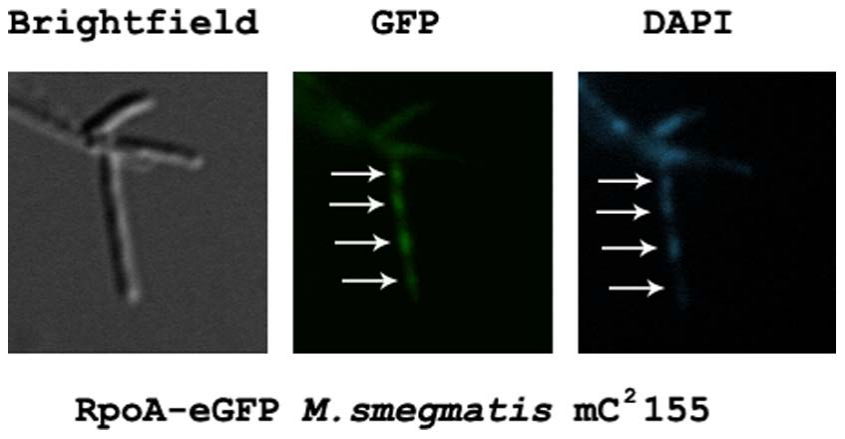
Subcellular localization of RpoA (MSMEG1524) fused to eGFP. Exponentially growing cells were induced with tetracycline for expression of the tagged target and counterstained with DAPI to visualize bacterial chromosomes. Arrows indicate RpoA co-localized with DNA.

In AP–MS studies, determining noise, false positives and false negatives is necessary to distinguish true interactions from contaminants. Sequential purification steps (e.g. tandem affinity purification) may decrease these unwanted results but at a risk of losing both weak and transient interactions. We analysed non-specifically binding proteins, commonly associated with all tested baits. We established average intensity values characteristic for each non-specifically binding protein for each of the 4 tags. The highest number of background proteins identified in all 8 proteins was observed with HA tag experiments (76 proteins) and the lowest with the eGFP tag (25 proteins). Non-specific binders for FLAG and ProtA experiments were 33 and 32, respectively (Fig. 4, Table S5). Moreover, we identified only 8 proteins in all 32 samples analysed, including 4 ribosomal proteins, 2 chaperons, a reductase and a transcription termination factor (Table S5). In addition, the samples were examined by SDS–PAGE and visualized by Coomassie staining (Fig. 2). The ProtA- and HA-tagged samples were enriched compared with the FLAG-tagged samples, correlating with the total number of identified proteins presented in Figure 3. The protein enrichment in eGFP tag experiments was higher than that in FLAG; however, considering the amount of background resin-bound proteins (Fig. 3B), an eGFP tag and respective resin provides a relatively low background and high specificity with high protein enrichment. We thus conclude that the eGFP tag combines the desirable features mentioned, offers easy ways to monitor binding efficiency by measuring GFP fluorescence (Table 2) and can be directly for localization experiments (Fig. 5). For further experiments, we selected the eGFP protein tag for practical application in mycobacterial proteomic experiments.

**Table 2:** Calculated binding efficiency based on eGFP fluorescence intensity detected in cell lysates vs. flow-through.

Locus name	cell lysate [fluorescence counts]	flow-through [fluorescence counts]	binding efficiency
MSMEG0358	5222428	978136	81%
MSMEG0752	2949152	336980	89%
MSMEG1524	10954260	4326100	61%
MSMEG1666	1897892	283148	85%
MSMEG2136	1763528	716720	59%
MSMEG3021	1125388	440396	61%
MSMEG3085	1021644	293248	71%
MSMEG3086	5509748	1315996	76%

### Protein complexes identified by AP–MS approaches

All proteins used as baits for affinity purification experiments in *M. smegmatis* purified on specific affinity resins and were identified as dominant proteins in the respective samples (Table S4). After removing the common contaminants as well as bead-specific contaminants, it was possible to observe complex formation for most of them. Some of them, such as the RNA polymerase complex, were predictable; others were completely novel, and they will need further studies for understanding the biological meaning of formation of such complexes in the mycobacterial cell. For instance, MSMEG_0358, annotated as the beta subunit of ribonucleoside diphosphate reductase, co-purified with considerable amounts of MSMEG_1960 and MSMEG_1961, both conserved hypotheticals, and MSMEG_1476, a signal peptide peptidase. These proteins were found exclusively in all purifications of MSMEG_0358, regardless of the tagging system. In addition, there was a substantial increase in the amount of MSMEG_6284, a cyclopropane-fatty-acyl-phospholipid synthase, in those samples. Another bait, MSMEG_1666, predicted to be an RNA polymerase sigma factor SigJ, specifically co-purified with MSMEG_4121, a GntR transcriptional regulator. Finally, MSMEG_3086, predicted to be triosephosphate isomerase (TpiA) when used as a bait, co-purified with MSMEG_3085, a phosphoglycerate kinase (Pgk) from the same operon but not vice versa. Pgk, on the other hand, was found to form a complex with MSMEG_4248, a 1-acylglycerol-3-phosphate O-acyltransferase, and MSMEG_2340, a hypothetical protein with limited similarity to isopentenyl pyrophosphate isomerase.

### Purification of DNA-directed RNA polymerase protein complex

Bacterial RNA polymerase is a well-characterized enzyme composed of 5 core subunits (α, α, β, β′ and ω) that bind accessory proteins such as sigma factors to form a functional holoenzyme [26], [27]. The structure of the *E. coli* core enzyme is available and importantly shares sequence similarity with mycobacterial homologues (α: 54,9%, β: 56,8%, β′: 55,0% and ω: 30,1%, *E. coli* to *M. smegmatis*). Because the structure and composition of the RNA polymerase complex is known, it is often used as a model for purifying protein complexes and thus RNA polymerase alpha subunit (RpoA; α) was selected as the target in our study. It allows to evaluate purification and accuracy of detection of the RNA polymerase subunits.

**Figure 6:**
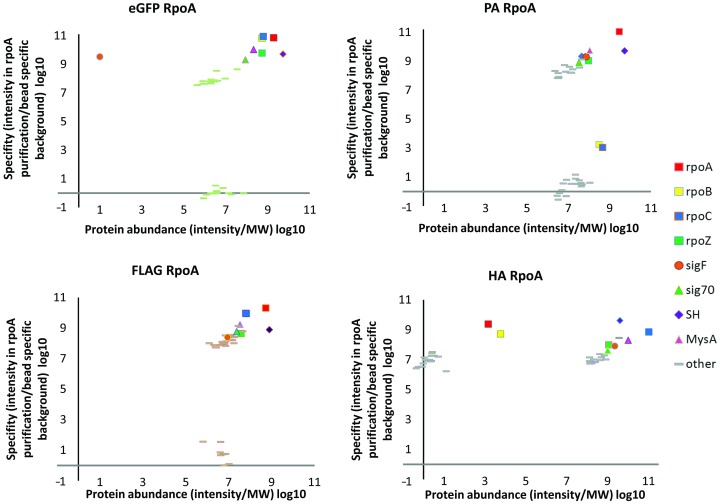
Semi-quantitative analysis of co-immunoprecipitation results using SH-quant peptide-tagged RpoA protein as bait. Points corresponding to subunits of the RPO complex are indicated with squares on scatter. Protein abundance was defined as the signal intensity calculated by MaxQuant software for each protein and divided by its molecular weight. Specificity was defined as the ratio of protein signal intensity measured during bait purification to background level. A protein was arbitrarily treated as background if it was found in all 8 purifications and its abundance was set as median intensity of values obtained in all purifications.

The 4 core subunits of RNA polymerase co-purified with RpoA fused with all 4 tested tags. Tagged RpoA with RpoB and C subunits were detected with high signal intensity (Fig. 6). The lowest intensity of subunits was found with FLAG tag. RpoZ, the smallest subunit of the holoenzyme, was detected with lowest intensity, but its sequence coverage was the same for all tags. Data presented in Figure 6 includes hits remaining after filtering out the first 40 proteins with highest intensity. Excluded proteins were classified as contaminants (Table S5). This method placed all known holoenzyme components in the top 10 of the hit list. The proteins with the highest abundance and with best enrichment *vs.* background were located in the top right-hand corner of each scatter. The abundance of RpoA was in agreement with that of SH-quant peptide, which is helpful for determining the number of RpoA molecules in each sample.

Because of its fast growth rate, well-established methods for genetic manipulation and biosafety level 1 requirements, *M. smegmatis* is one of the best organisms to study cellular mechanisms of its pathogenic cousin *M. tuberculosis*. However, *M. smegmatis* is a non-pathogenic mycobacterium (except in case extremely immunodeficient individuals), with a genome approximately 1.7 times larger than that of *M. tuberculosi*s. In order to use a model closer to *M. tuberculosis*, we tested our eGFP tag procedure in the *M. bovis* BCG Danish strain, which has an approximately 99.9% similarity to *M. tuberculosis* at the genetic level. Both *M. bovis* BCG and *M. tuberculosis* are member strains of the *M. tuberculosis* complex [28]. Thus, successful application of our method to BCG may suggest that the same approach can be used in virulent *M. tuberculosis* with only little modification. In addition, with high genetic similarity between the two, we expect little or no difference between native complexes, justifying *M. bovis* as a more optimal model organism to study protein–protein interactions for *M. tuberculosis*.

To test our method in *M. bovis* BCG, the coding sequence of *M. tuberculosis* RpoA (Rv3457c) was cloned into a pKW08-eGFP vector and transformed into *M. bovis* BCG by electroporation. The RNA polymerase protein complex was obtained by the same method used for *M. smegmatis*. In this experiment, we used both C-terminal tagging and N-terminal tagging to determine their influence on the purification outcome. In addition, we prepared a strain expressing eGPF-tagged SigB (Rv2710, identical with BCG_2723), one of the less abundant subunits found in the complex, to determine if the protein will be capable of pulling down the entire complex as well. The results presented in Table 3 show that all core subunits were favourably purified and detected by MS with high sequence coverage and intensity values. In all cases, we detected all RNA polymerase core subunits and 2 sigma factors, SigA (also referred to as MysA and RpoD) and SigB, in contrast to 4 sigma factors detected in *M. smegmatis*: SigA, sigma-70, sigma-F and SigB (Table S4). When SigB was used as a bait, it was purified as a dominating protein in the sample; however, it pooled down all the core RNA polymerase components and did not significantly affect the ratio between the other subunits.

**Table 3:** List of candidates identified after purification of *M. tuberculosis*-derived RpoA (Rv3457c, tagged with C-terminal or N-terminal eGFP) or SigB (Rv2710, tagged with C-terminal eGFP) expressed in *M. bovis* BCG.

Protein IDs	Description	Mol. weight [kDa]	Intensity		
			RpoA-CGFP	NGFP-RpoA	SigB-CGFP
BCG_0716	DNA-directed RNA polymerase beta subunit (RpoB)	129.2	5.17E+10	4.91E+10	1.40E+10
BCG_0717	DNA-directed RNA polymerase beta subunit (RpoC)	146.7	5.04E+10	4.85E+10	2.28E+10
**Rv3457c**/BCG_3522c	DNA-directed RNA polymerase alpha subunit (RpoA)	37.7	**3.89E+10**	**3.50E+10**	1.12E+10
BCG_1451	DNA-directed RNA polymerase omega subunit (RpoZ)	11.8	2.32E+09	2.31E+09	5.98E+08
BCG_2716	sigma factor SigA	57.8	1.14E+09	1.04E+09	5.15E+08
**Rv2710**/BCG_2723	sigma factor SigB	36.3	1.16E+08	8.50E+07	**1.08E+10**

Sequence coverage and intensity values are assigned by MaxQuant software. Intensity values for tagged subunits of DNA-dependent RNA polymerase are indicated in bold.

Because the eGFP tag can be applied to determine the subcellular localization of targeted proteins, we determined the RpoA-eGFP fusion localization within mycobacterial cells. RNA polymerase is known to exhibit affinity toward DNA; thus, it was not unusual to find that RpoA-eGFP co-localized with the mycobacterial chromosome (Fig. 5). Fusion protein localization may also suggest its functionality and that it may be involved with a holoenzyme. Moreover, induction of eGFP fusion protein production can be discerned under a microscope, an added useful feature.

### Analysis of the RNA polymerase subunit interaction using chemical cross-linking

Affinity purification is a standard method for analysing protein–protein interactions and topology of complexes by chemical cross-linking. Cross-linking converts non-covalent interactions between proteins surfaces into artificial covalent bonds. Cross-linking along with MS analysis can support modelling and aid in solving structures of complexes. Because the protein complex purification method based on eGFP protein fusion/resin has proven efficient with a relatively low background, we decided to test its application for cross-linking experiments. Because a 3D structure of the core RNA polymerase enzyme is available for *E. coli* (NCBI Molecular Modeling Database Accession Number 3LU0), and most of the mycobacterial components of this enzyme share high amino acid sequence similarity, we used protein cross-linking for establishing interactions between the homologous mycobacterial enzyme subunits. We employed the BS3 cross-linker that is reactive towards amine groups and is designed with a 11.4-Å spacer arm, which allows chemical cross-linking of 2 neighbouring lysine (K) residues and/or the N-terminal amino acid within reach of the spacer arm. Several cross-links were identified with pLink software (pFind Studio) (Table S6), and sample cross-links were then overlaid into the 3D structure model mentioned previously to assess proximity by measuring the distances between the *E. coli* amino acids in Chimera software. The products from cross-linking proved that mycobacterial core enzymes share high sequence and structure homology with their homologues in *E. coli* because many cross-links were separated by less than 20 Å and were positioned on the contact surface between 2 different protein subunits. Key examples were a cross-link between *M. smegmatis* RpoC K827 and RpoB K184, homologous to *E. coli* RpoC D751 and RpoB K164 (separated by 19.527 Å) and *M. smegmatis* RpoA K153 and RpoB K837, homologous to *E. coli* RpoA E162 and RpoB T927 (separated by 16.867 Å). Our experiments also confirmed the usefulness of cross-linking in assigning real interacting partners identified initially by AP–MS. Information obtained from cross-linking may indicate the structures of multiprotein complexes [29] and help to identify the contact surface between 2 proteins.

### Analysis of protein–protein complexes under changing growth conditions

Ideally, the method used in our study should translate to *M. tuberculosis* for investigating cellular processes. It is well established that the composition of various protein complexes may differ during various growth conditions or under stress. In this study, we decided to use UvrA, a protein involved in a process of DNA damage repair system (NER) and also well conserved between the bacterial species. UvrA is known to be in complex with UvrB, where a UvrA–UvrA dimer binds UvrB to form a DNA integrity-scanning complex, UvrA_2_B or UvrA_2_B_2_ [30]. The complex undergoes structural rearrangement and dissociates whenever it identifies helical distortions induced by a mismatched DNA sequence [31]. This enables recruitment of other proteins needed to complete repair. We expressed *M. tuberculosis* UvrA (*Rv1638*), which is identical to BCG_1676 from *M. bovis* BCG, in BCG to determine complex formation after DNA damage induction with UV light. We observed that UvrA was in complex with UvrB in cells, as expected. We were also able to monitor complex dissociation during DNA damage repair and re-association after the repair process was completed (Table 4). Polymerization of the newly synthesized DNA fragments was performed by DNA polymerase I, and we observed an enrichment of DNA polymerase I 5 min after induction of UV damage. Five minutes after UV irradiation, we observed the dissociation of the UvrAB complex, and 25 min later, the UvrAB complex was again detectable. This suggests that the kinetics of UvrA-B dissociation is similar to the kinetics observed in *E. coli* [30], despite the difference in doubling time between *E. coli* (20 min average) and *M. bovis* BCG (16–20 h). This result adds a dynamic capability to our method.

**Table 4:** Purification of protein interactors of UvrA after UV-induced DNA damage.

Protein IDs	Description	Mol. weight [kDa]	Intensity				
			UvrA 0’	UvrA 1’	UvrA 5’	UvrA 15’	UvrA 30’
**Rv1638**/BCG_1676	excinuclease ABC subunit A (UvrA)	106.2	**6.98E+08**	**5.11E+08**	**6.51E+08**	**2.21E+09**	**8.45E+09**
BCG_1671	excinuclease ABC subunit B (UvrB)	78.1	1.20E+07	5.97E+06			2.56E+06
BCG_3704c	DNA topoisomerase I	102.4	1.03E+06		2.64E+06		3.08E+06
BCG_3222c	putative DNA helicase II (UvrD2)	75.6	1.58E+06				1.60E+06
BCG_1667	DNA polymerase I	98.5			4.99E+06		

UvrA was eGFP-tagged at the C-terminus and expressed in *M. bovis* BCG. Samples were collected at 0, 1, 5, 15 and 30 min after exposure to UV light. Intensity values are given for DNA repair-associated proteins identified at listed time points only. Intensity values for tagged proteins are indicated in bold.

## Discussion

Affinity purification coupled with MS is used to identify proteins and their interacting partners. The first step is efficient purification of protein complexes with, ideally, no or little background. Optimizing this method to improve efficiency and breadth of interactions discovered would help in understanding the pathogen biology. Several affinity tags are now used to facilitate isolation of proteins with their partners. Based on the nature of the affinity tag and its target, we can distinguish several systems: protein-immobilized molecular ligand (hexahistidine metal) [32], protein–protein (calmodulin-binding peptide–calmodulin) [33] and subsystem protein–antibody (FLAG–anti-FLAG) [23]. A large number of affinity tags and specific binding resins are commercially available. Selecting the best for both protein bait and organism is indeed a key step for a successful experiment. Importantly, an accurately selected affinity tag allows proteins to be purified using generalized protocols [34], which is an important parameter in large-scale and high-throughput experiments. As Lichty et al. summarized, the ideal affinity tag (a) should be characterized by efficient, high-yield protein purification, (b) can be used with any protein without losing function, (c) can be placed at any position (C- or N-terminus), (d) can be used in any host or expression system, (e) can be easily used to detect the recombinant protein and (f) should bind and be eluted from an inexpensive resin [34]. Using affinity tags fused to a protein of interest allows production, isolation and accurate identification of interacting partners in the native system. Protein insolubility, conformation, stability, structural flexibility and purification yield and recovery are challenges that must be resolved in these experiments. Carefully chosen affinity tags and the relevant purification protocol, specific resin and elution method mitigate the aforementioned problems. In high-throughput experiments, affinity tag and purification method should be versatile, applicable and inexpensive. The most popular affinity tags and proteases used for tag removal have been detailed elsewhere [35]. We decided to test 4 different tags with 8 mycobacterial proteins expressed in a commonly used non-pathogenic laboratory strain *M. smegmatis* mc^2^155. All tags are a method for binding the protein to a resin with immobilized antibodies that recognize a specific epitope. We have shown that affinity tags can be used for protein purification from mycobacterial species, and interacting protein partners can be detected. The purity and background signal do vary. As described previously [34] and from our data, the highest purity with lowest quantity was obtained by using a FLAG tag. ProtA and HA tags yielded a large amount of interacting material, but with a high resin-specific background. We focused on the eGFP tag, which merges the high protein enrichment of ProtA and HA protocols with a relatively low background as seen with the FLAG tag.

In our study, the lowest background was detected in experiments using the GFP tag. In addition, one of the important features of this experimental setup is the ease of detection of tagged recombinant proteins. With the exception of GFP, all examined tags need special attention to visualize recombinant proteins within cells and to determine their subcellular localization and expression levels. Only cells expressing protein fused with eGFP can be directly used for microscopy. Our eGFP tag allows measurement of the binding efficiency of the tagged protein to the respective affinity resin (Table 2).

The eGFP tag is a full-length enhanced green fluorescent protein, which may impact structure or solubility of the tagged protein within cells. It is often detectable during overexpression of recombinant proteins when missfolded proteins aggregate and form inclusion bodies [36]. Therefore, expression of recombinant proteins in our system was relatively low and protein fusions were not visible as thick bands after gel electrophoresis of the cellular lysates, as it commonly is for overexpressed proteins (data not shown). When the 8 different proteins fused to eGFP were purified, aggregation was not detected during purification or in inclusion bodies by microscopy. Moreover, the eGFP tag can also be used for protein localization studies, allowing control of protein aggregation and localization screening of purified proteins.

Additional advantages of the eGFP tag included the ability to quickly and accurately control expression of the protein fused to eGFP, the high recovery ratio from the anti-GFP resin and the very low cost of purification. Cost is a critical parameter when an affinity tag and appropriate resin is selected, particularly for high-throughput experiments. We compared the price of purification for different affinity resins. In-house preparation of the anti-GFP affinity resin, as was done for this work, markedly decreases expenses (Table S7).

Deciphering the protein–protein interactions may be very helpful for improving our understanding of the biology and pathogenesis of *Mycobacterium*. To aid this quest, we compared 4 different affinity tags commonly used for affinity purification and evaluated their potential use in high-throughput experiments in mycobacterial model. Although, two-hybrid and three-hybrid systems have been used successfully [7], [9], [37], [38], similarly efficient assays need to be developed for use in the relevant native organism. Based on our data, we strongly advocate the use of eGFP-based affinity tags for protein purification and identification of protein–protein interactions in both small- and large-scale experiments in mycobacterial cells. Potential targets from the list of preys co-purified in the AP–MS experiments can be then confirmed by alternative techniques. Moreover, chemical cross-linking can be helpful for increasing the confidence of direct binary interactions between proteins and assigning the contact surfaces between them. This is particularly valuable for structural studies on complexes with known homology to already characterized complexes isolated from other organisms. This was the case for RNA polymerase in our studies. In addition to the numerous cross-links between *M. smegmatis* subunits that mapped to the *E. coli* model [39], there were a number of cross-links between the core RNA polymerase subunits and the sigma factors, providing additional information that can be useful for modelling of this essential large protein complex.

We used both C-terminal and N-terminal tagging of RpoA to determine tagging at which terminus allowed more efficient purification of the RNA polymerase complex from *M. bovis* BCG (Table 3). We found almost the same purification efficiency regardless of the placement of the eGFP tag. However, this certainly cannot be treated as absolutely true for every protein and some proteins will require a tag to be placed on a specific terminus to allow complex formation in living cells. This may be the reason for complex formation between the glycolitic enzymes TpiA and Pgk in only one combination, not the other. These enzymes were found to be closely linked in many other organisms. In *Thermotoga maritima,* they were found as covalently linked fusion proteins able to form a multimeric bifunctional complex [40]. It is highly possible that Pgk requires its C-terminus to be tag- free to be able to interact with and pull down TpiA in mycobacteria.

Using the nucleotide excision repair protein UvrA in *M. bovis* BCG model, we proved that AP–MS-based approaches are capable of detecting dynamic changes in protein complex formation under changing circumstances. Without the DNA damaging stimuli, UvrA was found to co-purify with substantial amounts of its partner UvrB and induction of DNA damage caused specific reactions to occur within the cell, with attempts to repair the damage caused by UV irradiation, resulting in temporary dissociation of the UvrAB complex. Our data also indicates the presence of possible additional factors in the damage-scanning mechanism: topoisomerase I (TopoI) and a DNA helicase II, annotated as UvrD2. However, additional studies will have to be conducted to understand the underlying mechanisms of such interactions. One possibility would be that the DNA integrity-scanning complex requires TopoI and DNA helicase II to respectively relax and unwind the DNA during scanning. In eukaryotes, it was shown that down modulation of topoisomerase I using antisense RNA inhibits repair of UV-induced lesions. The experiments show that TopoI is actively recruited onto genomic DNA following DNA damage by UV light, possibly acting during pre- or post-DNA damage processing [41]. Similar functions of topoisomerase I may be required for effective NER repair in *Mycobacteria* and possibly other prokaryotes. A previous study demonstrated the interaction between UvrA, UvrB and UvrD in *E. coli* using immunoprecipitation [42]. Our study provides additional evidence that these proteins form a complex in prokaryotic cells.

## Supporting Information

Table S1 **List of primers used in this study.**
(PDF)

Table S2 **Peptide identification details for AP-MS experiments.**
(XLSX)

Table S3 **Protein identification details for AP-MS experiments.**
(XLSX)

Table S4 **MaxQuant based semi-quantitative analysis of preys purified in AP-MS experiments.**
(XLSX)

Table S5 **List of prey proteins identified as common contaminants.**
(XLSX)

Table S6 **Cross-links between the subunits of RNA polymerase identified by pLINK software.**
(XLSX)

Table S7 **Comparison of expense of protein binding resins used in this study.**
(PDF)
