# Supplementary material for: Identification of Protein Partners in Mycobacteria Using a Single-Step Affinity Purification Method
Source: PLoS One. 2014 Mar 24;9(3):e91380. doi: 10.1371/journal.pone.0091380 (PMC3963859; doi:10.1371/journal.pone.0091380)
Supplement: Table S1 — List of primers used in this study. (PDF) [file pone.0091380.s001.pdf]

Supplemental Table S1

**Primer**	**sequence**
**F1**	GCTCGCTACTCTCATCGTGGAATCCTGACAGGATCCGCGATATCAAGCTTAAGGCCGCCGACATCACCTC
**ProtA R1**	CTCTAGGGTCCCCAATTAATTAGCTAAAGCTCAGGTCGACTTGCCGGCCGA
**FLAG R1**	CTCTAGGGTCCCCAATTAATTAGCTAAAGCTCACTTGTCGTCGTCGTCCT
**HA R1**	CTCTAGGGTCCCCAATTAATTAGCTAAAGCTCAGGCGTAGTCCGGCACGT
**GFP R1**	CTCTAGGGTCCCCAATTAATTAGCTAAAGCTCACTTGTACAGCTCGTCCA
**0752F**	GCTCGCTACTCTCATCGTGGAATCCTGACAGGATCCGTAC**GGAGGA**GTTGCCCATATGCCCATTGCCACACCTGAGG
**0752R**	GTACAGCGAGGTGATGTCGGCGGCCTTAAGCTTTGCCGCGGTGACGCTGCGTCCC
**2136F**	GCTCGCTACTCTCATCGTGGAATCCTGACAGGATCCGTAC**GGAGGA**GTTGCCCATATGTCAGCCAATCCGCGCGC
**2136R**	GTACAGCGAGGTGATGTCGGCGGCCTTAAGCTTCCCGATGACTGTATTCACCAC
**3021F**	GCTCGCTACTCTCATCGTGGAATCCTGACAGGATCCGTAC**GGAGGA**GTTGCCCATGTGTCCGACAGCTTGTTCGA
**3021R**	GTACAGCGAGGTGATGTCGGCGGCCTTAAGCTTGCGTCGCCTGCGGATGATGG
**0358F**	GCTCGCTACTCTCATCGTGGAATCCTGACAGGATCCGTAC**GGAGGA**GTTGCCCATATGACGCGGACACACTTCGA
**0358R**	GTACAGCGAGGTGATGTCGGCGGCCTTAAGCTTGTTCGCGGCGGCCGCGGCCG
**3085F**	GCTCGCTACTCTCATCGTGGAATCCTGACAGGATCCGTAC**GGAGGA**GTTGCCCATATGTCGGTCAAGACACTCGA
**3085R**	GTACAGCGAGGTGATGTCGGCGGCCTTAAGCTTCGACTCCAGTACTTGGATGC
**3086F**	GCTCGCTACTCTCATCGTGGAATCCTGACAGGATCCGTAC**GGAGGA**GTTGCCCATATGGCACGTAAGCCCCTCAT
**3086R**	GTACAGCGAGGTGATGTCGGCGGCCTTAAGCTTCGGAAGTGGTCCGCCCGCGGC
**1666F**	GCTCGCTACTCTCATCGTGGAATCCTGACAGGATCCGTAC**GGAGGA**GTTGCCCATGTGACCACCGCCGCGCGGAC
**1666R**	GTACAGCGAGGTGATGTCGGCGGCCTTAAGCTTTGGGCGTCTGACCGCGGTGC
**1524F**	GCTCGCTACTCTCATCGTGGAATCCTGACAGGATCCGTAC**GGAGGA**GTTGCCCATATGCTGATCTCTCAGCGTCC
**1524R**	GTACAGCGAGGTGATGTCGGCGGCCTTAAGCTTAAGCTGCTCGGTCTCGGCGT
