# Supplementary material for: Identification of Protein Partners in Mycobacteria Using a Single-Step Affinity Purification Method
Source: PLoS One. 2014 Mar 24;9(3):e91380. doi: 10.1371/journal.pone.0091380 (PMC3963859; doi:10.1371/journal.pone.0091380)
Supplement: Table S7 — Comparison of expense of protein binding resins used in this study. (PDF) [file pone.0091380.s007.pdf]

Tag	Size[aa]	Resin	Source	Binding capacity	Calculated cost/ 1mg of protein
Flag	28	Anti-Flag M2 Affinity Gel	Sigma-aldrich	>0.6mg/ml	above $500
HA	58	EZview^{TM} Red Anti-HA Affinity Gel	Sigma-aldrich	>0.4mg/ml	above $1000
ProtA	157	IgG Sepharose 6 Fast Flow	GE Healthcare	2mg/ml	$20
GFP	259	Anti-GFP	our lab	3.8mg/ml	$2.6
		GFP-Trap	ChromoTek	0.3mg/ml	above $1000
